# Anodic Polarization Behavior of X80 Steel in Na_2_SO_4_ Solution under High Potential and Current Density Conditions

**DOI:** 10.3390/ma12030394

**Published:** 2019-01-27

**Authors:** Runzhi Qin, Yanxia Du, Zhenchang Xu, Minxu Lu

**Affiliations:** Institute for Advanced Materials and Technology, University of Science and Technology Beijing, Beijing 100083, China; lcdcrtgp@hotmail.com (R.Q.); xuzhenchang0526@163.com (Z.X.); lumx123@sina.com (M.L.)

**Keywords:** X80 steel, HVDC interference, polarization behavior, corrosion, passivation, transpassivation

## Abstract

X80 steel has great risk of corrosion in high voltage direct current (HVDC) interference cases. In this study, the anodic polarization behavior of X80 steel under high potential and current density in Na_2_SO_4_ solution was investigated. The I × R drop was eliminated using current interrupt technique during the potentiodynamic measurement. Therefore, the real polarization curve was obtained. The corrosion behavior was investigated by galvanostatic polarization, scanning electron microscopy, and X-ray photoelectron spectroscopy. The results show a new form of passivation route. The steel dissolved actively below −0.388 V_SCE_, then became partly passivated from −0.388 to 1.448 V_SCE_, and fully passivated above 1.448 V_SCE_. The passive film was formed containing Fe_2_O_3_ and FeOOH, and resistant to SO_4_^2−^ ions. It not only blocked the direct dissolution of steel, but also facilitated oxygen evolution. The corrosion rates of steel samples decreased after the passivation.

## 1. Introduction

X80 carbon steel is used in pipelines worldwide. However, it may suffer various interferences and has the risk of corrosion. Recently, high voltage direct current (HVDC) systems have been developed rapidly around the world. Under fault and maintenance conditions, the system works in monopolar configuration and injects large amount of direct current (DC) into the soil, thus introducing DC interference to nearby buried pipelines. Therefore, the pipelines may have significant pipe-to-soil potential deviation and large leakage current density at coating holidays [1]. The HVDC interference is more severe than common DC interference. In some cases [2], the pipe-to-soil potential is as high as 304 V, and the leakage current density of the coupon is reported to be 0.049 A·cm^−2^. The HVDC cases have become a concern. However, the corrosion of pipeline steel under such high potential and current density conditions has been rarely studied. How to evaluate the corrosion risk has become an urgent issue in the industry.

To study the corrosion behavior, the anodic polarization curve is an important tool. The kinetics of corrosion can be obtained from the polarization results. Under low potential and current density conditions, the polarization behavior of carbon steel in soil simulation solutions has been extensively studied. Since SO_4_^2−^ ions are extensively and massively distributed in soil, the Na_2_SO_4_ solution is often used as a typical simulation solution in the laboratory. Some scholars [3,4,5,6] studied the polarization characteristics of carbon steel in Na_2_SO_4_ solution. The steel dissolved actively under the test condition (lower than 0.4 V_SCE_, or 0.010 A·cm^−2^). Although two theories on the dissolution of steel have been reported [7,8], it is well recognized that the whole steps could be summarized as the formation of Fe^2+^ ions [9]. Some studies have considered the effect of SO_4_^2−^ ions. Some researchers [10,11,12,13] tested the anodic polarization behavior of carbon steel in solutions with different concentration of SO_4_^2−^ ions. The results indicate that SO_4_^2−^ ions can facilitate the dissolution of steel. Thus, SO_4_^2−^ ions are proved to be the most aggressive anions. However, prior experiments were carried out in narrow potential/current density ranges and thus did not satisfy the HVDC interference cases. The polarization characteristics of pipe steel in Na_2_SO_4_ solution should be studied further. 

Moreover, the IR drop is a difficult issue in potential measurement. The directly measured apparent potential *E_on_* contains IR drop, the voltage drop of electrolyte between the working electrode and reference electrode [14]. In HVDC interference cases, with the great increase in current density, the IR drop becomes a significant error. Although some theoretical calculations and optimization have been carried out [15], the IR drop still cannot be completely eliminated in experiments. Besides, the IR drop compensation of the commercial electrochemical workstation does not work well under high voltage/current density conditions. To obtain the precise polarization potential, the current step method was proposed, and the potential-time response and substance concentration were analyzed [16]. On this basis, some researchers [17,18,19,20] tested the IR drop in various environments using the current interrupt method and subtracted it from the apparent potential. Therefore, the feasibility and effectiveness of IR drop elimination under high potential and current density conditions deserve more attention.

In this study, the polarization behavior of X80 steel under high potential and current density in Na_2_SO_4_ solution was studied by potentiodynamic polarization. The current interrupt method was used to test and eliminate the IR drop. Combined with galvanostatic polarization and product characterization, the corrosion process and corrosion rate were analyzed, and a mechanism model in the test range was proposed. This study provides a reference to better understand the corrosion and protection of pipe steel in large DC interference cases.

## 2. Materials and Methods

### 2.1. Materials and Solution

The test material was API X80 steel; it was cut from an in-service pipeline system. The chemical constitution in weight percentage (wt%) was: 0.07 C, 0.21 Si, 1.61 Mn, 0.12 Ni, 0.14 Cu, 0.041 Nb, 0.012 Ti, 0.0025 S, 0.0081 P, 0.13 Mo, and Fe balance. The specimen was embedded in paraffin wax, exposing the working area of 1 cm^2^. Before each experiment, the working electrode surface was abraded with 360, 600, 800, and 1000 grit silicon carbide emery papers and ultrasonically cleaned with acetone, ethyl alcohol, and deionized water and then dried under cold dry air.

The test solution was 4 g·L^−1^ Na_2_SO_4_; this was made from analytical-grade reagents and deionized water.

All tests were carried out at room temperature of 25 ± 2 °C and relative humidity of 30 ± 2%.

### 2.2. Potentiodynamic Polarization Measurement and IR Drop Elimination

**Figure 1:**
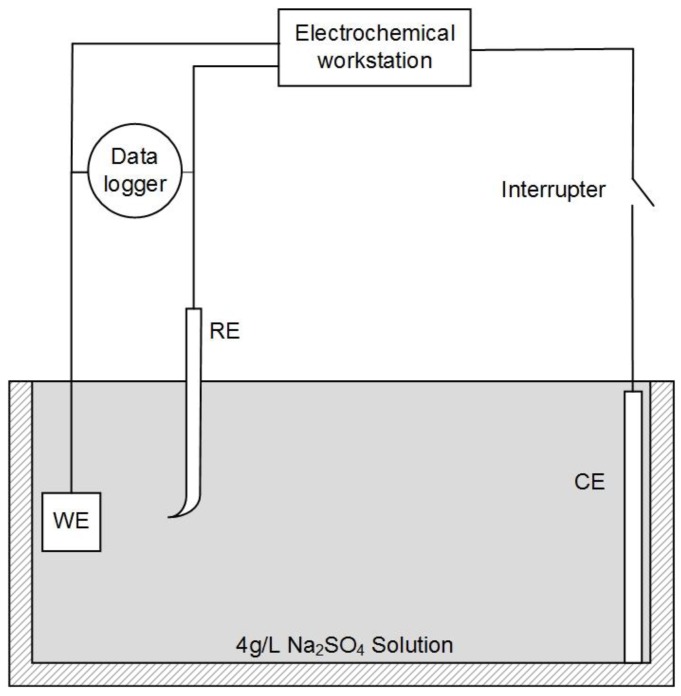
Schematic diagram of the electrochemical test circuit combined with the current interrupt method.

A three-electrode system was used for the experiments, as shown in Figure 1. X80 steel coupons, a Pt electrode, and a saturated calomel electrode (SCE) were used as the working electrode (WE), counter electrode (CE), and reference electrode (RE), respectively. In our experiments, all electrochemical potentials were expressed with respect to SCE. An electrochemical workstation (Gamry Reference 3000, Gamry, Warminster, UK) was used for electrochemical measurements. When the open-circuit potential of WE was stable, the potentiodynamic curve was measured at a potential scanning rate of 1 mV·s^−1^ from open circuit potential (OCP) to +7 V. This test range could fully cover the highest leakage current density in HVDC cases [2].

During the potentiodynamic measurement, the current interrupt method was used for IR drop evaluation and elimination. An interrupter was installed in the CE branch to interrupt the external current periodically. A 1000-Hz data logger (DL-1, Tinker & Rasor, San Bernardino, CA, USA) was used to record the potential of WE during the interruption. Then, the IR drop was calculated from the potential–time transient.

### 2.3. Corrosion Rate and Product Characterization

Galvanostatic polarization measurements were carried out to determine the corrosion rate and characterize the products. The samples were polarized under a constant current of 0.001, 0.010, 0.020, 0.030, 0.040, 0.050, 0.080, 0.100, and 0.150 A·cm^−2^ for 1 h. After the galvanostatic polarization, the surfaces of the specimens were rinsed with deionized water and dried with cold dry air.

Based on the polarization results, samples with currents of 0.010, 0.030, and 0.080 A·cm^−2^ were selected for product characterization. The surface morphologies of corrosion scales were observed by scanning electron microscopy (SEM, LEO-1450, Zeiss, Jena, Gernamy), and their composition was investigated by X-ray photoelectron spectroscopy (XPS, AXIS ULTRADLD, Kratos, Manchester, UK).

For all samples, according to the ISO 8407: 2010 standard [21], the rust was eliminated by successive cleaning in hydrochloric acid aqueous solution (500 mL deionized water + 500 mL concentrated hydrochloric acid + 5 g hexamethylenetetramine). The coupons were washed with acetone and dried by air blast. Then, the corrosion rate was calculated.

## 3. Results

### 3.1. IR Drop Elimination

In the polarization measurement, the apparent potential *E_on_* contains a certain amount of IR drop. Their relationship can be described as follows:

*E_on_* = *E_p_* + *IR_u_* (1)

where *E_p_* is the real polarization potential, *I* is the external current flowing through WE, and *R_u_* is the uncompensated resistance of electrolyte between the working electrode and reference electrode [14]. In this paper, the word “real” is used to describe the potential corrected by IR drop elimination. The real potential was more precise than the apparent potential, as discussed below.

Generally, when the current is interrupted, the measured potential *E(t)* exponentially decays with time [20]:

(2) $$E\left(t\right)=E_{0}+\left(E_{p}-E_{0}\right)\times e^{-t/\tau }$$

where *E*_0_ is the eventual potential after infinite time and *τ* is the time constant. Theoretically, *E_p_* can be described as *E*(0), which can be measured at the exact moment of the current interruption. Some scholars [18,19] used oscilloscopes to find *E*(0). However, in practical operations, it is difficult to obtain the accurate *E*(0) because of the limit of fast interruption and datalogger. 

**Figure 2:**
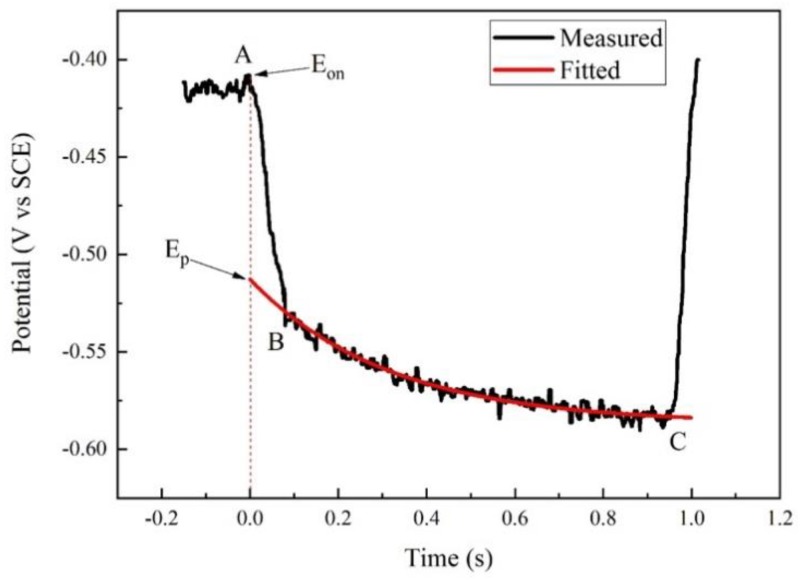
Transient potential–time curve during one interruption of polarization of X80 steel in 4 g·L^−1^ Na_2_SO_4_ solution.

During the potentiodynamic measurement in our experiment, the current was interrupted periodically. At each interruption, the potential was recorded using a high-speed datalogger. Taking one interruption process as an example, the transient potential–time curve is shown in Figure 2. The interrupter started to work at time A, but the complete interruption of circuit occurred at time B. To obtain the accurate *E_p_* at time A, the potential values were fitted and extended reversely, as shown in the red curve. The cross-point of the fitted curve and time A was *E_p_*, which was regarded as the real polarization potential. Accordingly, the IR drop was obtained by subtracting *E_p_* from *E_on_*.

**Figure 3:**
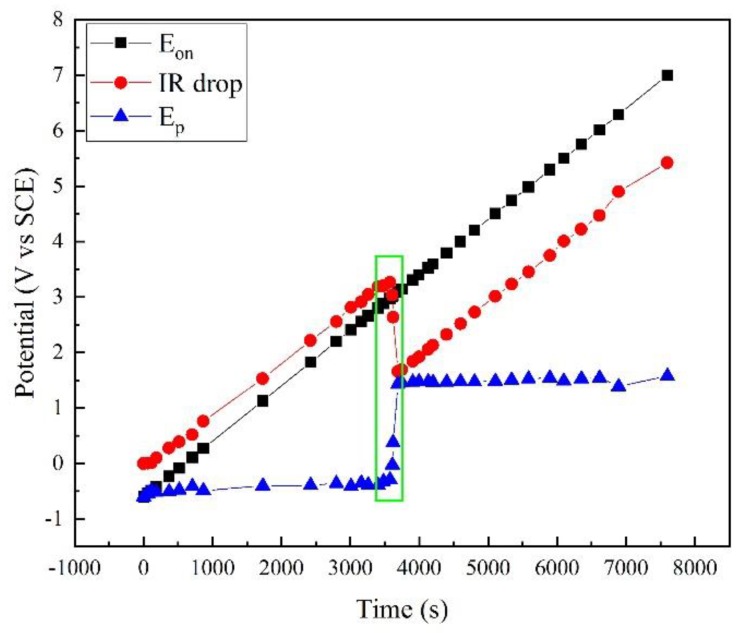
Comparison of *E_on_*, *E_p_*, and IR drop in polarization measurement of X80 steel in 4 g·L^−1^ Na_2_SO_4_ solution.

Based on this method, the polarization potential *E_p_* was calculated, as shown in Figure 3, together with the apparent potential *E_on_* and the IR drop. Clearly, *E_on_* linearly increased as programmed. The IR drop increased with *E_on_*. However, a sudden drop occurred at ~3500 s. Correspondingly, a sudden rise occurred in the *E_p_* curve. At other times, *E_p_* slightly increased with time.

Figure 3 shows that the apparent potential *E_on_* contains a large amount of IR drop, and the real polarization information was covered by this error. For example, when *E_on_* was 7.002 V, *E_p_* was only 1.577 V. The IR drop was 5.425 V, and the error was 77.5%. In fact, the ordinary potential control was not reliable under high potential/current density conditions because of the IR drop. Some traditional test methods such as potentiostatic polarization and cyclic voltammetry are no longer available.

### 3.2. Potentiodynamic Polarization Results

**Figure 4:**
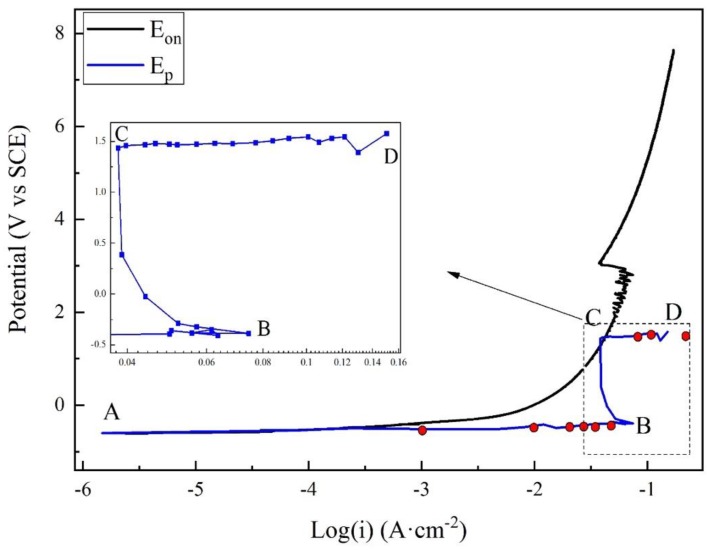
Comparison of apparent and real polarization curves for X80 steel in 4 g·L^−1^ Na_2_SO_4_ solution.

**Figure 5:**
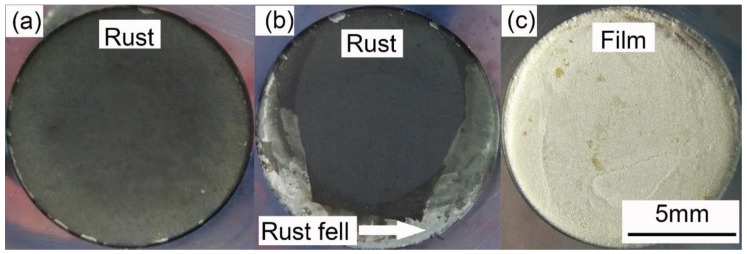
Macro-morphologies of sample surfaces in: Stage 1 (A–B of Figure 4) (**a**); Stage 2 (B–C of Figure 4) (**b**); and Stage 3 (C–D of Figure 4) (**c**).

Figure 4 presents the anodic potentiodynamic polarization curves of X80 steel in 4 g·L^−1^ Na_2_SO_4_ solution. The *E_on_* curve was drawn as the black line. The *E_p_* curve, which corresponded to all the *E_p_* values, was drawn as the blue line, and the characteristic points were marked from A to D. Both the curves can be separated into three stages. The macro-morphologies of the sample surface in different stages are shown in Figure 5.

Stage 1: A–B. Point A was the open-circuit potential of X80 sample, −0.598 V. When the current density was lower than 0.001 A·cm^−2^, both *E_on_* and *E_p_* had a linear relationship with the logarithm of current density, described as Tafel behavior. The Tafel slope was 75.9 mV·dec^−1^ for *E_on_* curve, and 43.2 mV·dec^−1^ for *E_p_* curve. After 0.001 A·cm^−2^, *E_on_* increased more quickly than the logarithm of current density. Clearly, the reason was the growth of IR drop, as displayed in Figure 3. However, the *E_p_* curve still followed the Tafel behavior. In this stage of experiment, the specimen gradually lost its gloss, and a layer of corrosion products appeared on its surface. First, the products were gray. Then, their color turned to black and became darker, as shown in Figure 5a. The solution became straw yellow, probably indicating the diffusion of Fe^3+^ ions.

Stage 2: B–C. When the current density reached 0.073 A·cm^−2^ at Point B, *E_p_* was −0.388 V. Point B was a turning point of the curve. After Point B, *E_p_* sharply increased, while the current density decreased. It indicated some new electrochemical reactions. This was also the reason for the decline of IR drop shown in Figure 3. At some positions, the black rust began to fall off from the steel, exposing the bare metal, as shown in Figure 5b. Moreover, massive bubbles gushed from such positions. With the progress in experiment, more and more rust dropped.

Stage 3: C–D. Point C was another turning point of the curve, where the current density decreased to 0.038 A·cm^−2^, and *E_p_* was −1.448 V. Both *E_on_* and *E_p_* continuously increased beyond Point C; the current density started to increase again until the limit of the electrochemical workstation. For the *E_p_* curve, a new Tafel relationship with the current density was obtained. The new Tafel slope was 74.6 mV·dec^−1^. In this stage, the black rust was completely shed, exposing the metal surface. There was a layer of certain film on the sample surface, as shown in Figure 5c. The film will be further discussed below.

As shown in Figure 4, the apparent polarization curve deviates from the Tafel behavior with increasing current density. This was due to IR drop instead of the limit of mass transfer of reactant/product [16]. For the real polarization curve, two turning points indicate new electrochemical processes, as discussed below.

### 3.3. Corrosion Product Characterization

To study the reaction process of X80 steel in 4 g·L^−1^ Na_2_SO_4_ solution in different periods, galvanostatic polarization measurements were applied to the samples. The values of constant current density are shown as the red points in Figure 4. For each galvanostatic polarization, the potential–time curves are misleading because of the large IR drop. Therefore, only the product characterization and corrosion rate analysis are used here.

**Figure 6:**
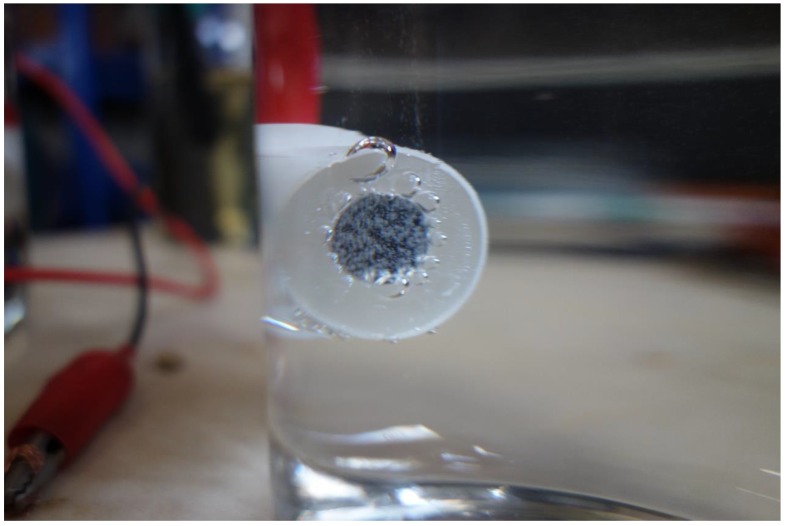
Oxygen evolution on the sample surface during the galvanostatic polarization test with a current density of 0.100 A·cm^−2^.

In the experiments, when the current density was below 0.080 A·cm^−2^, corrosion occurred, and rust was formed. When the current density was higher than 0.080 A·cm^−2^, some gas bubbles were produced on the sample surface, as shown in Figure 6. The gas was collected and detected to be O_2_, because of combustion-supporting characteristic. Moreover, no obvious corrosion rust was observed on the sample surface in this situation.

**Figure 7:**
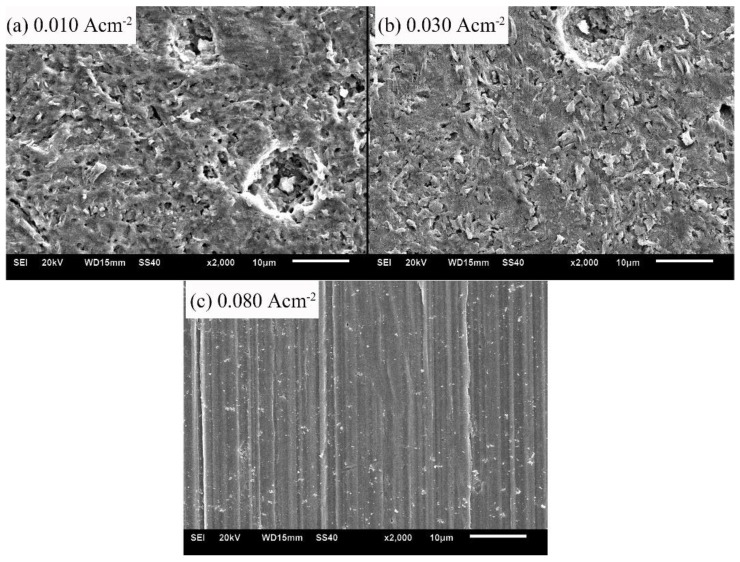
Micro-morphologies of sample surfaces after the galvanostatic polarization test of: 0.010 A·cm^−2^ (**a**); 0.030 A·cm^−2^ (**b**); and 0.080 A·cm^−2^ (**c**).

The micro-morphologies of samples after the galvanostatic polarization of 0.010, 0.030, and 0.080 A·cm^−2^ are shown in Figure 7. With low current densities (0.010 and 0.030 A·cm^−2^), the corrosion products were bar-shaped crystals. The crystals accumulated to a flat layer, but the layer was loose and porous. With a high current density of 0.080 A·cm^−2^, the product layer was neat and even, indicating that the corrosion product was tiny and densely crystallized.

**Figure 8:**
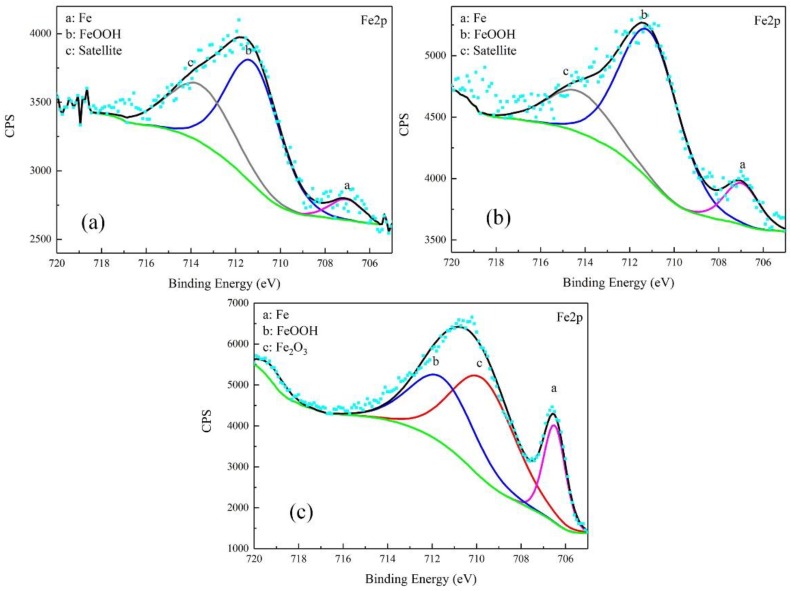
Detailed XPS spectra of Fe2p of corrosion products on samples after galvanostatic polarization of: 0.010 A·cm^−2^ (**a**); 0.030 A·cm^−2^ (**b**); and 0.080 A·cm^−2^ (**c**).

**Figure 9:**
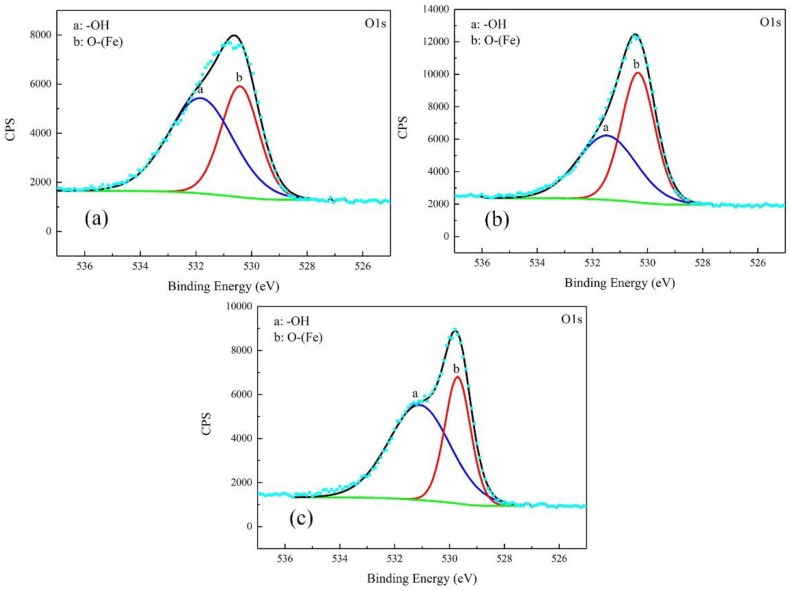
Detailed XPS spectra of O1s of corrosion products on the samples after galvanostatic polarization of: 0.010 A·cm^−2^ (**a**); 0.030 A·cm^−2^ (**b**); and 0.080 (**c**).

The corrosion products were characterized by XPS. The results were free of S. The high-resolution spectra and decomposition of Fe 2p and O 1s curves are shown in Figure 8 and Figure 9. 

For the products obtained after the galvanostatic polarization test of 0.010 A·cm^−2^, three major peaks were observed at 707.1, 711.2, and 713.6 eV in the Fe 2p curve. They were assigned to Fe matrix, FeOOH, and the satellite of Fe^3+^, respectively [22,23,24]. The O 1s curve can be decomposed to two major peaks at 530.1 eV and 531.8 eV. This was attributed to metal oxides and hydroxides [25,26,27]. The peaks in the O 1s and Fe 2p curves in the case of 0.030 A·cm^−2^ were similar, indicating the same type of corrosion products. 

In the case of 0.080 A·cm^−2^, three major peaks appeared at 706.5 eV, 709.7 eV, and 711.5 eV in the Fe 2p curve, corresponding to the Fe matrix, Fe_2_O_3_, and FeOOH, respectively. The peaks at 529.7 eV and 531.1 eV in O 1s curve were assigned to metal oxides and hydroxides. The peak of Fe matrix was high, indicating that the product was as thin as several nanometers.

### 3.4. Corrosion Rates

**Figure 10:**
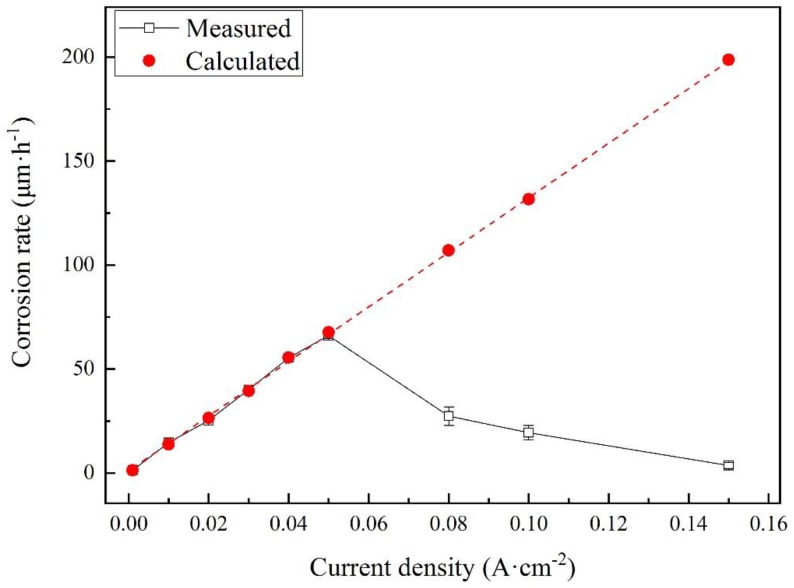
Comparison of measured and calculated corrosion rates for X80 steel after different galvanostatic polarization tests in 4 g·L^−1^ Na_2_SO_4_ solution.

The corrosion rates of the samples were measured after different galvanostatic polarization tests. For each current density, the experiments were repeated at least three times. The measured corrosion rates are shown as black hollow dots in Figure 10. Besides, the corrosion rates were calculated using Faraday’s law and the well-accepted corrosion reaction:

Fe = Fe^2+^ + 2e (3)

The calculated results are shown as red dots. The real corrosion rates linearly increased and fit the calculated values well before 0.080 A·cm^−2^. The highest corrosion rate was measured to be 66.1 μm·h^−1^ at 0.050 A·cm^−2^. However, the corrosion rates had a huge drop at 0.080 A·cm^−2^ and then decreased with the current density. The results indicate that some new reactions occurred, inhibiting the corrosion of steel.

## 4. Discussion

According to the polarization curves, product characterization, and corrosion rate results, the polarization of X80 steel was separated into three regions, namely dissolution, transition, and transpassivation:

(1) Dissolution

The range of dissolution region was Stage 1, from −0.598 to −0.513 V. In this region, *E_p_* and the current density fit the Tafel relationship, and the measured corrosion rates satisfied the calculated values based on Equation (3). Carbon steel actively dissolved in various electrolytes, only in a narrow range of potential/current density [2,4,5,6,7,11,12,13]. In this experiment, the range was extended to 0.073 A·cm^−2^. The active dissolution mechanism of carbon steel in Na_2_SO_4_ solution has been fully studied [7]. The first step of corrosion was well recognized as the formation of Fe^2+^ ions, as shown in Equation (3).

Then, the Fe^2+^ ions participated in the formation of rust, as shown in Equation (4). Based on the SEM morphology analyses reported in the literature, this type of rust looked more like cigar-shaped γ-FeOOH (akaganeite) [28] than spherical 𝛼-FeOOH (goethite) [29,30] or flowery 𝛾-FeOOH (lepidocrocite) [28,31]. This rust layer was porous and unable to block further dissolution.

4Fe^2+^ + 4H_2_O + 2O_2_ = 4FeOOH + 8H^+^ (4)

(2) Transpassivation

The polarization curve of Stage 3, as shown in Figure 4, satisfied the new Tafel relationship, and the real polarized potential was almost 2 V higher. In this region, the SEM and XPS showed a thin and dense film on the sample surface. Moreover, a large amount of oxygen bubbled, and the corrosion rate decreased. These phenomena indicated that the steel was in a passivation state, and the main composition of the passive film was Fe_2_O_3_ and FeOOH. Some researchers believed that the passive film of carbon steel usually had a double-layer structure [32]. The outer layer was FeOOH [33,34,35], and the inner layer was Fe_2_O_3_, most probably γ-Fe_2_O_3_ (maghemite) [36]. Under different test conditions, the passive film in our experiments was consistent with the literature. The formation of the film can be described as follows:

2Fe^2+^ + 3H_2_O = Fe_2_O_3_ + 6H^+^ + 2e (5)

Fe^2+^ + 2H_2_O = FeOOH + 3H^+^ + e (6)

This passive film was protective. It inhibited the further dissolution of steel and hindered the formation and development of rust. Therefore, the corrosion rates remained at a low level. At the same time, with the increase in current density, the oxygen evolution reaction (OER) became more and more violent, consuming a large amount of current density (Equation (7)). Therefore, the corrosion rates were lower in high current density conditions.

2H_2_O = O_2_↑ + 4H^+^ + 4e (7)

(3) Transition 

In Figure 4, Stage 2 was in the transition region. The polarized potential showed a sudden rise at Point B, indicating the start of passivation reactions. However, the process was fast in this region. It was difficult to maintain the samples at this region in galvanostatic polarization.

In the transition region, notably, the passivation did not occur evenly in the entire working area. Instead, it started from some local positions with the drop of rust, as shown in Figure 5b. It can be inferred that the rust and film were mutually exclusive. The film was not transformed by the rust but directly formed between the steel substance and rust. At that time, it should be noted that the measured potential was a mixed potential, which was still below the potential of OER. However, at some places, the local potential exceeded the potential of OER, therefore OER took place. The gas bubbles filled the interface and broke the structure of the rust, pushing the rust down. As the potentiodynamic measurement progressed, the area of passive film increased, and the rust continued to fall. Finally, the rust fell off completely, and the steel reached the transpassivation region.

**Figure 11:**
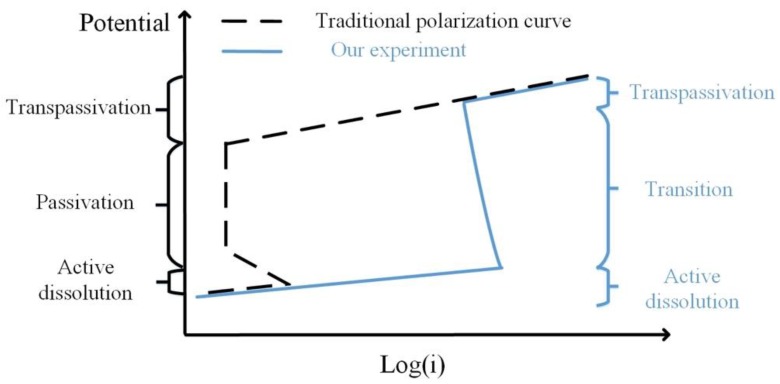
Schematic diagram of the traditional polarization curve and our experiment result.

The passivation in this experiment was quite different from ordinary passivation. Figure 11 shows a comparison of the two curves. In the traditional potentiodynamic process, the passivation reaction evenly occurs on the sample surface with a large decrease in current density (usually in the level of 10^−6^ A·cm^−2^ [32]). However, in our experiments, the passivation reaction started locally and developed to the transpassivation state. Thus, the current density was higher than the traditional cases. Besides, no such decline of current density was observed. The transition state was more like a mixture of dissolution and transpassivation. This passive mode has been rarely reported before. 

**Figure 12:**
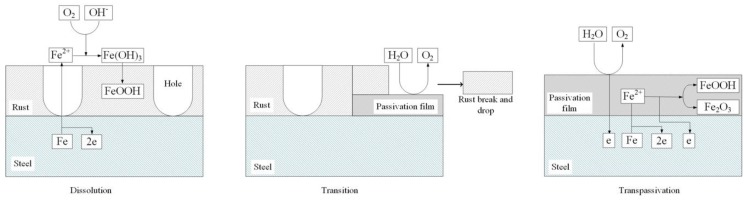
Reaction model of anodic polarization behavior of X80 steel in 4 g·L^−1^ Na_2_SO_4_ solution.

Based on the results and analysis mentioned above, the dissolution–transition–transpassivation model of X80 steel in Na_2_SO_4_ solution is proposed, as shown in Figure 12. In the dissolution region, the steel dissolved, and rust formed on the sample surface. In the transition region, passivation reaction started locally, and rust started to fall. In the transpassivation region, the passive film covered the sample surface, and OER took place strongly. 

It is well accepted that the SO_4_^2−^ ions can facilitate dissolution. In some cases, the addition of a slight amount of SO_4_^2−^ ions could break the passive film and increase the corrosion rate [37,38,39,40,41,42]. However, our experiments proved that this passive film of X80 steel was resistant to SO_4_^2−^ ions. Moreover, the corrosion rates decreased because of the passive film. These results may provide useful information for corrosion evaluation and protection in high voltage/large current density interference cases.

## 5. Conclusions

During the anodic polarization measurement of X80 steel in the Na_2_SO_4_ solution, the current interrupt method was used. The IR drop was calculated by fitting the potential–time transient of interruption. The IR drop increased with current density, and it was the main error in the potential measurement under high potential/large current density conditions. 

The real polarization curve was obtained by IR drop elimination. A new form of passivation was observed. In the test range, the anodic process showed three types of characteristics: dissolution, transition, and transpassivation. The active dissolution region was below −0.388 V, where the samples dissolved, and the rust of FeOOH was formed. The transition region was from −0.388 V to 1.448 V. The passivation reaction occurred locally on the sample surface, and the rust started to fell off because of OER. When the polarized potential was above 1.448 V, the steel reached the transpassivation region. The passive film consisted of Fe_2_O_3_ and FeOOH and showed resistance to the SO_4_^2−^ ions. Strong OER occurred on the film surface. Based on the experimental results and analysis, a mechanism model of the anodic polarization process of X80 steel in Na_2_SO_4_ solution was proposed.

In the activation region, the corrosion rates of X80 steel were directly proportional to the anodic current density. However, in the transition and transpassivation regions, the passive film hindered the direct dissolution of steel. Besides, a large amount of current density was consumed in OER. Therefore, the corrosion rates of X80 steel significantly decreased.
